# Supplementary material for: Integration of Metabolomics and Transcriptomics to Explore Dynamic Alterations in Fruit Color and Quality in ‘Comte de Paris’ Pineapples during Ripening Processes
Source: Int J Mol Sci. 2023 Nov 16;24(22):16384. doi: 10.3390/ijms242216384 (PMC10671212; doi:10.3390/ijms242216384)
Supplement: Supplementary file 1 [file ijms-24-16384-s001.zip › ijms-2658327-supplementary.pdf]

Table S1. Primer used for quantitative real-time PCR analysis.

**Name**	**Accession number**	**Forward primer (5′–3′)**	**Reverse primer ((5′–3′)**
Acactin	HQ148720	CTGGCCTACGTGGCACTTGACTT	CACTTCTGGGCAGCGGAACCTTT
AcCCR	Aco015695.1	TTCCTCCATCCAAAGTTCCC	CCGACAGCCGCACAAGA
*Ac*CCR	Aco026870.1	ACGCCGCAAACCAAGAT	TTTACGGTCCTCCTTCCACA
AcCCR	Aco005397.1	GATTTGGAAGCGAAAGCAGC	GCGGTCACGGGCATAGTT
AcCAD	Aco001032.1	TCGGGATGAAAGTTACGG	CATTTGCTCAGGGTTGC
AcCOMT	Aco000946.1	CTACATTCATTACCACGAGA	CGAAAGCTGGGATAGTT
AcCOMT	Aco000839.1	TTACAAGACAATCGCTACA	TAGGGATAGAGTTGATAGTAGA
Ac4CL	Aco019975.1	TCGTTCAGGGCTATGGTATG	GTTTCAAAGTCAAGGCGGTA
AcLAC	Aco015409.1	TTCGCTGGGTTGTTGGC	TCGTCCGTTTACTGCTGTGAT
AcPOD	Aco002506.1	AAGGGTGAAGGCTAAAGTGG	GCATTGGGTGATGGCAGT
AcPOD	Aco008456.1	GGAGGAGGTTGAGCTATTTG	TGCTTTGGGACAGGTTTT
AcF3H	Aco027900.1	ATGGTGAAGGCAATGGGG	CGTTGGGGTCTTTGTGGC
AcDFR	Aco006769.1	GAGCACCAACGGTCAGAGT	AACAACAAGAGGTGGGATGA
AcANR	Aco010710.1	CAATCCTACGGTTGTGCG	CATCGAGCGGAGTCCAG
AcGST	Aco001260.1	ACGAGGACTTAGGCAACA	CAGGATGACCAGCGACT

Table S2. Summary of transcriptome data for pineapple fruit at YF, MF, and FMF stages.

Raw sequences and assembly statistics	
Raw reads (paired-end)	46284834 - 74558864
Clean reads (paired-end)	44474662 - 72092510
Retention rate	98.37% - 98.83%
Q20	98.11% - 98.29%
GC content percentage	50.41% - 53.45%
Rate of cleans reads pairs mapped to the reference genome	62.00% - 75.17%
